# Epidemiology, Clinical Features, and Unusual Complications of Norovirus Infection in Taiwan: What We Know after Rotavirus Vaccines

**DOI:** 10.3390/pathogens11040451

**Published:** 2022-04-09

**Authors:** Meng-Che Lu, Sheng-Chieh Lin, Yi-Hsiang Hsu, Shih-Yen Chen

**Affiliations:** 1 Division of Allergy, Asthma and Immunology, Department of Pediatrics, Shuang Ho Hospital, Taipei Medical University, New Taipei City 23561, Taiwan; superrayworld@gmail.com (M.-C.L.); jacklinbox@tmu.edu.tw (S.-C.L.); 2 Department of Pediatrics, School of Medicine, College of Medicine, Taipei Medical University, Taipei city 11031, Taiwan; 3 Beth Israel Deaconess Medical Center and Harvard Medical School, Boston, MA 02215, USA; yihsianghsu@hsl.harvard.edu; 4 Broad Institute of MIT and Harvard, Cambridge, MA 02142, USA; 5 Division of Pediatric Gastroenterology and Hepatology, Department of Pediatrics, Shuang Ho Hospital, Taipei Medical University, New Taipei City 23561, Taiwan

**Keywords:** norovirus, epidemiology, gastroenteritis, clinical features, complications

## Abstract

Noroviruses (NoVs) are one of the emerging and rapidly spreading groups of pathogens threatening human health. A reduction in sporadic NoV infections was noted following the start of the COVID-19 pandemic, but the return of NoV gastroenteritis during the COVID-19 pandemic has been noted recently. Research in recent years has shown that different virus strains are associated with different clinical characteristics; moreover, there is a paucity of research into extraintestinal or unusual complications that may be associated with NoV. The genomic diversity of circulating NoVs is also complex and may vary significantly. Therefore, this short narrative review focuses on sharing the Taiwan experience of NoV infection including epidemiology, clinical features, and complications following suboptimal rotavirus immunization in Taiwan (after October 2006). We also highlight the unusual complications associated with NoV infections and the impacts of NoV infection during the COVID-19 pandemic in the literature for possible future research directions. To conclude, further research is needed to quantify the burden of NoV across the spectrum of disease severity in Taiwan. The evidence of the connection between NoV and the unusual complications is still lacking.

## 1. Introduction

Norovirus (NoV) is a nonenveloped virus with a single-stranded, positive-sense, polyadenylated RNA genome that encodes three major open reading frames (ORFs) [1]. ORF1 encodes a large polyprotein that is cleaved by the viral proteinase into six nonstructural proteins, which are involved in NoV replication in host cells. ORF2 encodes the major viral capsid protein (VP) 1 [2], whereas ORF3 encodes VP2, a minor capsid protein involved in viral packaging [3]. The capsid protein consists of a shell (S) domain encoded by conserved genetic lineages and two protruding (P) subdomains: the relatively conserved P1 and highly variable P2 subdomains. The P1 subdomain plays a role in viral-particle stability, whereas the outermost P2 subdomain contains epitopes recognized by neutralizing antibodies and the clefts required for binding to human histo-blood group antigen (HBGA) receptors [4,5,6]. Recombination at the junction of ORF1 and ORF 2 can result in the emergence of a novel viral strain, similar to other RNA viruses [7].

NoVs are highly diverse viruses that can be genetically grouped into 10 genogroups from GI to GX. Only the GI, GII, GIV, GVIII, and GIX genogroups can infect humans, and the GII genogroup is the most prevalent [8,9]. Variants (subgenotypes) are determined by sequence analysis of highly variable regions of the ORF2 and named according to the location and year they were first described [9]. NoV is an emerging pathogen that causes enteric infections in humans and has diverse clinical presentations. Genetic diversity of NoV indicates its rapid evolution with genotype polymorphism. NoV variants related to severe infection and complications have been reported sporadically, and the rapid genetic evolution of NoV is believed to drive changes in its clinical manifestations [10,11,12]. In addition to gastrointestinal (GI) symptoms, NoV infection is associated with several complications, including benign infantile convulsion [13], encephalopathy [14,15,16], necrotizing enterocolitis [17,18], dysregulation of the enteric nervous and immune system [19,20], exacerbation of inflammatory bowel disease [21], and chronic, serious outcomes in immunocompromised patients [22,23]. In Taiwan, human NoVs are the common cause of gastroenteritis outbreaks and the major cause of both all-age-group diarrhea and foodborne disease [24,25,26]. After suboptimal use of rotavirus vaccines in the private sector (after October 2006) in Taiwan, the increasing prevalence of severe NoV gastroenteritis was found. A study in Taiwan revealed a trend of reduced rotavirus to NoV ratio in prevalence, from 40% to 11.6% (2008 to 2009), 25.3% to 18.2% (2009 to 2010), and 22.2% to 25.9% (2010 to 2011) [27]. This short narrative review will focus on Taiwan experiences of NoV infection including epidemiology, clinical features, and complications. We also highlight the unusual complications associated with NoV infections beyond acute gastroenteritis and the impacts of NoV infection under the COVID-19 pandemic in the literature for possible future research directions.

## 2. Methods

We conducted a narrative review of the literature about the epidemiology, clinical features, and unusual complications of NoV infection in Taiwan. We searched original or review articles published after 1 January 1980, that related to NoV infection. PubMed was used for searching up to 25 January 2022, without language restriction. Search terms were “norovirus,” “clinical features,” “complications,” “vaccine,” and “rotavirus vaccine.” References listed within bibliographies of the retrieved records and personal files of the authors were also considered. Our team screened all identified titles and abstracts, and then full-text publications were reviewed for eligibility. The review period for eligible publications was from 4 October 2021, to 1 January 2022. We subjectively decided which studies to include according to our clinical experience in Taiwan. Any uncertainties were resolved through team discussions and consensus.

The suboptimal use of rotavirus vaccines in the private sector in Taiwan was after October 2006. We defined and divided the period into the early postvaccine period (January 2007 to December 2011) and the late postvaccine (January 2012 to December 2016) periods. The data shown in Table 1 are pediatric patients (<5 years old) with acute gastroenteritis, defined as acute-onset non-bloody watery diarrhea. The patients were all hospitalized at Chang Gung Children’s Hospital, a medical center located in northern Taiwan. The patients were enrolled without interest conflicts and medical difference and regardless of age, gender, ethnicity, social-economic level, and hospitalization ward. The distributions of enteric pathogens in the two periods were compared. The samples were analyzed with the Pearson χ^2^ test, and *p* values of <0.05 were considered to indicate statistical significance. All tests were performed using the SAS software (ver.8 for Windows).

## 3. The Molecular Epidemiology, Evolution and Recombination in Taiwan

### 3.1. The Change of Genotypes in Taiwan

In most areas of the world, GII.4 NoV predominated throughout the decade, and its role persisted even after rotavirus vaccine implementation [29,30]. During the era of post-rotavirus vaccination, considerable genetic diversity among NoVs was discovered worldwide. Although the genotypes varied among studies in different areas, GII.4 was still the most common over the recent 10 years [31,32,33,34,35,36,37,38,39]. The increasing prevalence of NoV infection is also associated with the recent change in global genotype distribution such as GII.4 [11,29,31,40,41]. Even from 2015 to 2020, based on the pooled prevalence of NoV infection among 120,531 children with gastroenteritis from 45 countries, the most common genotypes were still GII.4 (59.3%) and GII.3 (14.9%) according to the meta-analysis by Farahmand M. et al. [42].

In Taiwan, the significant increase in severe NoV gastroenteritis in children after suboptimal rotavirus immunization suggests an important role for the NoV genotype [27]. The data in Taiwan indicate that NoV GII.4 activity was high at the time of rotavirus vaccination introduction (late 2006 to early 2007), which was during a global epidemic. GII.4 NoV causes significant morbidity associated with gastroenteritis in infants [31,32,43]. The major genotypes GII.4 and GII.3 are responsible for the majority of NoV-associated gastroenteritis cases among children [25]. In early 2015, genotypes other than GII.4 were also evident such as GII.17, which emerged as it did in many other Asian countries [44]. However, a recent report in Taiwan from 2015 to 2019 showed that NoV GI.3 was the most common genotype detected in outbreaks of NoV gastroenteritis. These NoV outbreaks in Taiwan mainly occurred in preschool to secondary school students (0–18 years) [45]. The prevalence of NoV GI is also higher than that in previous studies undertaken other countries such as China, South Korea, and Thailand [46,47,48]. Since the next dominant genotype in Taiwan is unknown, continued surveillance for NoV typing is critical to monitor the emergence and impact of these GI.3 strains and other possible new NoV strains. Further research is required to discover or establish a model to predict possible NoV outbreaks.

### 3.2. The Change of Variants in Taiwan

The genomic diversity of circulating NoVs is complex and may vary by geographic area, age, or other variables. Previously predominant strains can be replaced by the emergence of new NoV variants every few years [42,49].

According to the reports in Taiwan, after rotavirus vaccination introduction (late 2006 to early 2007), the predominance of NoV GII.4 (75.7%) was similar to that reported in the United States (CaliciNet) (72%) and Finland (76%) [25,50,51]. The number of GII.4 2010 (the major subtype during the postvaccine period in Taiwan) infections was greater than that of GII.4 2006b [27]. The crucial amino acids in the human HBGA binding interfaces (interface I—S343, T344, R345, and H347; interface II—D374; interface III—C440–Y443) in VP1 are conserved in all GII.4 NoVs isolated in northern Taiwan. Therefore, the variations in the amino acid sequences adjacent to the amino acids that affect the physicochemical properties of the HBGA-binding interfaces of VP1 between the GII.4 2006b and 2010 subtypes are located in protruding domain 2 (P2 domain). The seven amino acids (T340, D357, A368, D372, N378, N412, and I413) in GII.4 2010 differing from those in GII.4 2006b are located in the P2-domain loops, which form the outer surfaces of the domain [52].

From late-2011, GII.4 2012 variants caused another community outbreak in northern Taiwan [53]. GII.4 2012 variants have been emerging since mid-2011 in northern Taiwan. These variants accounted for 54.7% of NoV infections, which is similar to the high rate of the GII.4 Sydney infections previously reported by the Centers for Disease Control and Prevention in the USA (53%) in children and adults [53,54]. The pandemic GII.4 variant, Sydney 2012—a new variant from a GII.e–GII.4 2010 recombination event—played an even more dominant role and became a prevalent strain worldwide [25,55,56,57,58,59,60]. The GII.4 variant, Sydney 2012, soon became the predominant circulating strain globally, replacing most other GII.4 variants [42]. In Taiwan, the GII.4 2012b variant, which evolved from GII.4 2006b, remained prevalent until NoV GI.3 outbreaks around 2018. It is seen that the GI.3 genotype accelerated in variation and showed transmission dynamics; variants are not identical to their parent strain in 2018, according to the research by Chiu et al. [25,45]. Due to multiple occurrences of different NoV lineages and their rapid evolution, timely interventions are critical to understand the complexity of norovirus gene variation and to monitor emerging norovirus strains.

## 4. Viral Gastroenteritis in Taiwan: From Rotavirus to Norovirus

In undeveloped countries, viral gastroenteritis is a notable cause of death in infants. In industrialized countries, gastroenteritis with diarrhea is usually self-limiting but morbidity is high among children and the elderly [61,62]. NoV and rotavirus are the most common viral causes of gastroenteritis. NoVs usually cause a short-term, self-limited illness with diarrhea and vomiting; however, the emerging new variants caused a distinct clinical syndrome of acute gastroenteritis with severe fever and a high prevalence of abdominal pain and intestinal hemorrhage, mimicking bacterial enterocolitis [25]. The estimated prevalence of NoV in acute gastroenteritis patients in developing countries was 17% [63]. Although the mortality rate caused by NoV or rotavirus in developed countries is low, there are still sporadic outbreaks that tend to occur in densely populated places [64].

**Figure 1:**
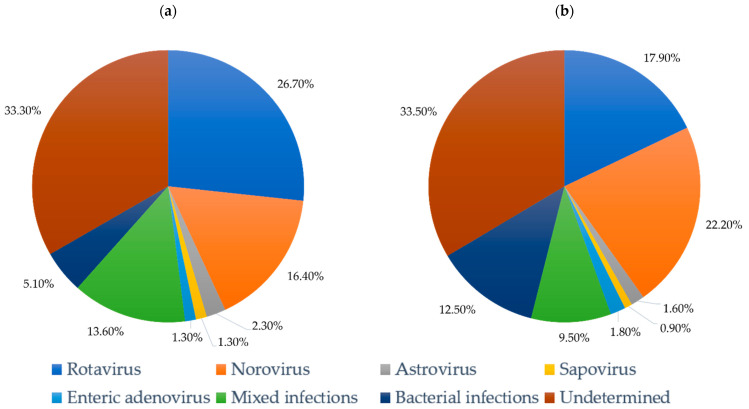
The distribution of enteric pathogen detection among hospitalized AGE children under 5 years old after rotavirus vaccine implementation in (**a**) 2007–2011, the early postvaccine period; (**b**) 2012–2016, the late postvaccine period. The data were originally from the research by Yu et al. [28].

In Taiwan, there is a long-term impact of rotavirus vaccines on acute gastroenteritis. A 10-year study in hospitalized children under 5 years old in Taiwan, which divided the study period into the early postvaccine period and the late postvaccine period, showed a significantly decreased prevalence of rotavirus infection (*p* = 0.002) and a significantly increased prevalence of NoV (*p* = 0.034) and enteric bacterial infections (*p* < 0.001) [28]. The comparison of pathogen prevalence between the early and late postvaccine periods and the distribution of enteric pathogens detection in hospitalized AGE children under 5 years old after rotavirus vaccine implementation from 2007 to 2016 in Northern Taiwan is shown in Table 1 and Figure 1.

The burden of rotavirus disease remained important though its association with severe gastroenteritis was decreased. After introduction of the rotavirus vaccine, the prevalence of rotavirus in hospitalized children with acute gastroenteritis reduced from 26.7% in the early postvaccine period to 17.9% in the late postvaccine period. Sustained reduction of more than 30% was achieved versus the prevalence in the prevaccine period (27.8%; 2004–2006). The significantly increased NoV infection rates have replaced rotavirus as the leading cause of child hospitalizations for gastroenteritis [27,28,65,66]. In 2021, a study by Ballard et al. [67] in the US showed that NoV is well-recognized as the leading cause of pediatric gastroenteritis in settings with universal rotavirus vaccination. In Taiwan, active and continued studies on the burden of NoV are needed. There is a relative paucity of research into quantifying the burden of NoV across the spectrum of disease severity.

## 5. Associated Complications Related to Norovirus Infection

**Figure 2:**
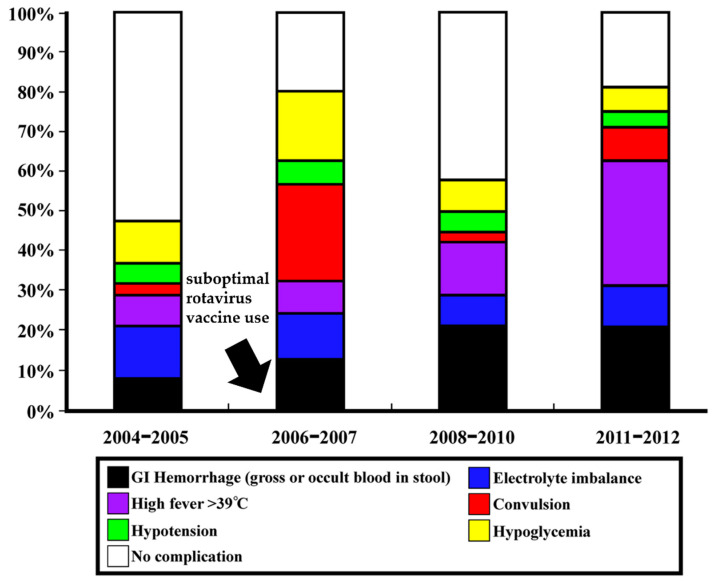
Complications in different periods of NoV outbreaks in northern Taiwan before and after suboptimal rotavirus vaccine introduction from late 2006 (highlight by arrow). The figure has been modified and originated from the research by Wang et al. [26].

According to the volunteer challenge studies, the asymptomatic infections were estimated at 32.1% [68]. In Taiwan, from winter 2004 to winter 2005, the most common complications were electrolyte imbalance (14.3%) and hypoglycemia (11.4%). After the rotavirus vaccination introduction (from late 2006), the most common complications were convulsive disorder (28%) and hypoglycemia (20%) from winter 2006 to winter 2007. In the 2008/09/10 winters, major complications included GI hemorrhage (22.2%) and prominent hyperthermia (13.8%). From winter 2011 to winter 2012, hyperthermia (34.9%) and GI hemorrhage (23.3%) were the most prominent complications caused by NoV infection [26]. Figure 2 demonstrates the overall complications of NoV infection in different periods in northern Taiwan.

Although the specific NoV subgenotypes related to severe infection and complications were only sporadically reported, the rapid genetic evolution of NoV is believed to be the main factor driving the changing clinical manifestations in infected patients. In the 2006/2007 winters, the NoV subgenotype or variant GII.4 Den_Haag_2006b (40%) caused the most complications of convulsion (67.9%) in infected children. The major subgenotype in the 2008/09/10 winters, GII.4 2010 (New Orleans) (48.5%), caused GI hemorrhage (37.5%). Furthermore, GII.4 2012 subgenotypes (56.8%), the predominant NoV strains in the 2011/2012 winter, caused more high fever (57.1%) and GI hemorrhage (33.3%) [26]. Research in northern Taiwan for the clinical relevance and genotypes of circulating NoVs found that patients infected by NoV GII.4 2006b had a higher frequency of diarrhea, longer duration of diarrhea, and more frequent hypoglycemia and electrolyte imbalance compared with those with gastroenteritis caused by NoV GII.4 2010 [51].

Studies in the last 15 years have reported that NoV infection is also associated with a range of sequelae and complications other than gastroenteritis. A recently developed model for NoV infection contributed critical knowledge for extraintestinal or unusual complications. There is still a paucity of research into associated complications in Taiwan. The details of possible associated sequelae or complications with NoV are given below.

### 5.1. Chronic Gastroenteritis

Chronic NoV gastroenteritis is the major sequela of NoV infection in primary immune deficient and oncologic patients, transplant recipients, and those infected with the human immunodeficiency virus [22,23,69]. Chronic NoV gastroenteritis can present specific clinical challenges in immunocompromised patients. Immunosuppressed patients experience prolonged fecal NoV shedding [70]. NoV diarrhea in cancer patients can contribute to decreased quality of life, interruption of cancer care, malnutrition, and altered mucosal barrier function [71]; in primary immune deficient groups, it is associated with protracted diarrhea, weight loss, and the requirement of parenteral nutrition [72]. Transplant recipients have a similar risk for developing recurrent or chronic infection with NoV as other groups [73]. Immunocompromised patients with chronic NoV infection only have limited options beyond supportive care. For cancer or transplant patients, though only few data support this strategy, immunosuppression should be decreased [74]. There is currently no effective antiviral regimen for chronic NoV infections. Several treatments have been attempted, including serum-derived human immunoglobulin, Nitazoxanide, antiviral agents such as Favipiravir, adoptive T-cell therapy, fecal microbial transplantation, probiotics, and complex microbial communities with glycans with affinity to NoV [75,76,77,78,79]. Further studies are needed to determine if those strategies could be suitable therapies to treat chronic NoV.

### 5.2. Necrotizing Enterocolitis

The gut microbiota composition during infancy or early childhood is variable, dynamic, and influenced by both prenatal and postnatal factors [80]. Many studies have shown that intestinal viral infections may be associated with severe illness such as hemorrhagic enteritis and even necrotizing enterocolitis (NEC) [17,18,81,82]. Although studies mentioned previously indicated an episodic association of enteric viruses in NEC, one study by Skeath et al. [83] in 2016 for 17 NEC infants without detection of any related viruses may suggest that viral etiology is unlikely to be causative for most sporadic forms of NEC. Understanding of the mechanisms in microbiota-immunity-infectious agent axis is necessary to define potential preventive or therapeutic tools against significant infections in children [80].

### 5.3. Irritable Bowel Syndrome

Irritable bowel syndrome (IBS) is a prolonged and disabling functional GI syndrome that affects 9–23% of the population across the world [84]. Acute gastroenteritis is a risk factor for postinfectious irritable bowel syndrome. The pathophysiology of IBS includes several possible mechanisms, such as visceral hypersensitivity, irregular gut motility, abnormal brain–gut relations, and the role of infectious agents; a systematic review showed similar risks for bacterial pathogens and indicated that studies are still limited for viral and parasitic pathogens [85]. There are already many studies showing that a number of pathogens correspond with the IBS disease, such as *Clostridium difficile*, *Escherichia coli*, *Campylobacter jejuni*, *Chlamydia trachomatis*, *Helicobacter pylori*, *Pseudomonas aeruginosa*, *Salmonella* spp., and *Shigella* spp.; viruses, particularly NoV; and even parasites [86]. Innate and adaptive immune mechanisms are involved in control of human NoV infection. NoVs may serve as viral triggers for IBS in some environmental and genetic contexts and also indicate that the microbiota may be important for NoV pathogenesis [87]. This still needs further work for understanding interactions between NoVs and bacteria in the gut and understanding the phenotypic outcomes from NoV mutation and evolution.

### 5.4. Inflammatory Bowel Disease

Inflammatory bowel disease (IBD) includes ulcerative colitis and Crohn’s disease. The burden of IBD is rising globally, with substantial variation in levels and trends of the disease in different countries and regions. There are an estimated 6.8 million IBD cases worldwide, and the prevalence rates range from 79.5 to 84.3 per 100,000 individuals [88]. Although there is some evidence that NoV is involved in the dysregulation of enteric nervous and immune system [19,20] or exacerbation of IBD [21], IBD is influenced by gut microbiota, complex inflammatory pathways, and cell–virus interactions. IBD is also affected by confounding variables such as diet, age, smoking, or psychological stress, as demonstrated in monozygotic twins [89]. While the effect of cytomegalovirus infection on IBD has been confirmed, the role of other viruses in IBD pathogenesis or exacerbation of disease symptoms is still controversial [90,91]. A recent study by Tarris et al. [92] found a strong expression of sialylated Lewis a and Lewis x antigens and human NoV viral-like particles (VLPs) binding in the absence of ABO antigen expression in IBD regenerative mucosa. Further studies are required to explore the implications of NoV in the impairment of epithelial repair and dysregulation of inflammatory pathways [92]. Recent findings regarding human NoV replication in intestinal enteroids and organoids are promising [93,94]. The use of organoids derived from IBD patients might be useful to investigate the virus–host interactions and genetic responses related to NoV [95].

### 5.5. Convulsions and Encephalopathy

Some patients with acute gastroenteritis develop convulsions that may be febrile or afebrile convulsions [96]. Specific enteric pathogens have been linked to convulsions in children, such as rotavirus, NoV, *Campylobacter*, and *Shigella* [13,14,15,97,98,99,100]. The change in the prevalence of convulsions related to gastroenteritis might be associated with rotavirus vaccination [101]. Several reports have shown that NoV related to mild gastroenteritis causes convulsions without fever, severe dehydration, electrolyte imbalance, and hypoglycemia [13,102,103,104]. Severe central nervous system complications such as meningitis, encephalitis, and encephalopathy have been also described [14,15,16,105,106,107]. A study in the era of post-rotavirus vaccination in Taiwan for molecular epidemiology of NoV gastroenteritis showed that seizures occurred in 20.9% of children with NoV infection. GII.4 Den_Haag_2006b (42.3%) and GII.4 Sydney 2012 (19.2%) were major variants correlated with convulsions. Compared with GII.4 Den_Haag_2006b, the GII.4 Sydney 2012-associated convulsions had similar manifestations except without significant winter preponderance. The NoV infection with convulsions had less febrile course, specific genotype (GII.4) infections, and shorter instances of vomiting [52]. More studies and continuous surveillance are essential for uncommon convulsions associated with emerging NoV strain infections.

#### 5.5.1. Benign Convulsions with Mild Gastroenteritis

Benign convulsions with mild gastroenteritis (CwG) were first reported by Morooka in 1982 [108]. CwG is characterized by afebrile convulsions within 5 days of acute viral gastroenteritis in previously healthy infants and children between the ages of 6 months and 3 years without electrolyte balance or abnormal blood sugar level or abnormal result of cerebrospinal fluid analysis. Several reports have broadened the range of definitions for children aged <6 years with CwG [103,104,109,110,111,112,113,114]. Convulsions in CwG are mostly generalized, usually clustered, and last less than 5 min. Although clustering seizures frequently occur in the acute phase of CwG, the prognosis is good and long-term treatment is usually not required [109].

After rotavirus vaccination, some changes in epidemiology and clinical characteristics occurred in NoV-associated CwG [103,104,112,113,115]. The ratio of NoV-associated CwG to NoV gastroenteritis and that of rotavirus-associated CwG to rotavirus gastroenteritis were different among studies [113,116]. One nationwide dataset from health insurance reviews and associated services in South Korea showed that the prevalence of rotavirus-associated CwG did not change (0.013–0.024%) but the annual prevalence of NoV-associated CwG is increasing by 1.790 times each year from 0.00001% [112,113]. However, these studies have limitations as they use diagnostic codes without compiling data directly from patient charts. Therefore, further studies are necessary to determine the actual prevalence after rotavirus vaccination.

There are many reports on the pathophysiological mechanism of CwG but most of them are for rotavirus [117,118,119,120,121,122]. Several studies have attempted to identify the mechanisms of NoV-associated CwG. NoV penetration into the gastrointestinal tract and RNA in the serum and cerebrospinal fluid have been observed [106,123]. There is still a paucity of studies on the pathophysiology of NoV-associated CwG.

#### 5.5.2. Encephalopathy/Encephalitis

Encephalitis is associated with significant mortality despite intensive care [124]. There were few reports or case series, mainly from Japan, related to NoV-associated encephalopathy or encephalitis [16,106,107,125]. A nationwide survey of NoV-associated encephalitis/encephalopathy in Japan showed that the outcome of children with NoV-associated encephalitis was poor. Poor prognosis included early onset of neurological symptoms, an elevated serum creatinine level, and an abnormal blood glucose level [16]. Elevated concentrations of cerebrospinal fluid interleukin-6, interleukin-10, interferon-γ, and tumor necrosis factor-α indicated that the encephalopathy may be related to hypercytokinemia rather than direct viral invasion [125]. Future studies are required since there is no effective treatment and the definite pathophysiology of NoV-associated encephalitis/encephalopathy is still unknown.

### 5.6. Other Possible Associated Extraintestinal Complications

There have been reports that NoV infection is associated with Guillain–Barre syndrome and its variant Miller Fisher syndrome [126,127]. Although it is still unclear if NoV is the one of the antecedent infections in Guillain–Barre syndrome, it might be linked to the molecular mimicry [128]. As NoV serology is usually unavailable in routine clinical practice, further studies related to this response are needed.

## 6. The Impact of COVID-19 Pandemic on Norovirus Disease

The COVID-19 (SARS-CoV-2) pandemic emerged from a cluster of patients with pneumonia of an unknown cause linked to a seafood wholesale market in Wuhan, China, in December 2019 [129]. With personal hygiene awareness and strict public health or nonpharmaceutical interventions including social restrictions, physical distancing, and international and domestic border closures for the COVID-19 pandemic, decreased NoV infection was noted across the globe [130]. NoV infections dropped 49% in the United States post-COVID-19 lockdown in December 2020 [131]. A decrease in NoV infections was also noted in Germany (reaching near 0%) and Australia (declining by 49.0%) in 2020 [132,133]. Although a sustained reduction in NoV was temporally associated with COVID-19 mitigation processes, positivity rates for other common enteric pathogens, especially bacterial enteritis, were only intermittently reduced [134].

Since health systems have been focused on the COVID-19 pandemic, there have been reports describing underreporting of various diseases around the world, including NoV infection [135]. Recent studies from China showed the return of NoV-related acute gastroenteritis during the COVID-19 pandemic in cities with multiple nonpharmaceutical interventions during winter 2020/2021 [136,137]. Similar outbreaks were also reported in the United Kingdom (UK), caused mainly by NoV in educational and care home settings, with prevalence around 61% and 34%, respectively [138]. The initial benefit of COVID-19 countermeasures that reduced the viral gastroenteritis burden is unsustainable.

It is important to maintain epidemiological surveillance in Taiwan for viral gastroenteritis and increase awareness of NoV during the COVID-19 pandemic. Additional analysis on the route of transmission of cases would be helpful [136].

## 7. Discussion

Dominant NoV strains evolve divergently over time. The genomic diversity of circulating NoVs is complex and may vary by geographic area, age, or other variables. In Taiwan, from 2015, NoV GI.3 has become the most common genotype in outbreaks of NoV gastroenteritis. The prevalence of NoV GI is higher than that in previous studies undertaken other countries such as China, South Korea, and Thailand [46,47,48]. Even though NoVs usually cause a short-term, self-limited illness, the emerging new variants caused a distinct clinical syndrome of acute gastroenteritis with severe symptoms mimicking bacterial enterocolitis. The rapid genetic evolution of NoV is believed to be the main factor driving the changing clinical manifestations in infected patients. Complications from severe acute gastroenteritis by NoV do exist, but there are still some possible associated extraintestinal or unusual complications such as chronic gastroenteritis, necrotizing enterocolitis, irritable bowel syndrome, inflammatory bowel syndrome, convulsions, encephalopathy/encephalitis, and even Guillain–Barre syndrome. Further research is required to provide evidence of the connection between NoV and those extraintestinal or unusual complications. The mechanisms in the microbiota-immunity-infectious agent axis, virus–host interactions, and genetic responses related to NoV may be possible key points.

Even though a sustained reduction in NoV has been temporally associated with COVID-19 mitigation processes, it is important to maintain epidemiological surveillance for viral gastroenteritis and increase awareness of NoV outbreaks during the COVID-19 pandemic. According to the experience in China and the UK [136,137,138], NoV infection outbreak may spread very easily and quickly around the nation during the COVID-19 pandemic. It is essential to set an active and continued surveillance network in NoV typing to monitor the emergence of possible new NoV strains and the change in clinical features. Further multiyear studies are needed to quantify the burden of NoV across the spectrum of disease severity.

## 8. Conclusions and Limitations

The evidence of the connection between NoV and the range of unusual complications is still lacking. The mechanisms in the microbiota-immunity-infectious agent axis, virus–host interactions, and genetic responses related to NoV may be possible key points. Further research is needed to quantify the burden of NoV across the spectrum of disease severity in Taiwan. Finally, there is a limitation in this review. As it is a narrative review focused on the Taiwan experience, the nature of the method may be too subjective, especially regarding the determination of which studies to include and the conclusions drawn.

## Figures and Tables

**Table 1:** Comparison of pathogen prevalence between the early and late postvaccine periods in hospitalized AGE children under 5 years old after rotavirus vaccine implementation. The dataset was from the research of Yu et al. [28].

Pathogens	2007–2011 (Early Postvaccine Period) (*n* = 396)	2012–2016 (Late Postvaccine Period) (*n* = 441)	*p*-Value
Rotavirus (*n*, %)	106 (26.7%)	79 (17.9%)	0.002 *
Norovirus (*n*, %)	65 (16.4%)	98 (22.2%)	0.034 *
Astrovirus (*n*, %)	9 (2.3%)	7 (1.6%)	0.427
Sapovirus (*n*, %)	5 (1.3%)	4 (0.9%)	0.742
Enteric adenovirus (*n*, %)	5 (1.3%)	8 (1.8%)	0.519
Mixed infections (*n*, %)	54 (13.6%)	42 (9.5%)	0.062
Bacterial infections (*n*, %)	20 (5.1%)	55 (12.5%)	<0.001 *
Undetermined (*n*, %)	132 (33.3%)	33.5%	0.945

* Indicates statistical significance.

## Data Availability

Not applicable.
